# Supplementary material for: Trends in prevalence and correlates of tobacco use among school-going adolescents in Bhutan: A secondary data analysis of the 2004–2019 Global Youth Tobacco Survey
Source: PLOS Glob Public Health. 2024 Jul 26;4(7):e0003544. doi: 10.1371/journal.pgph.0003544 (PMC11280154; doi:10.1371/journal.pgph.0003544)
Supplement: S1 Data — (PDF) [file pgph.0003544.s001.pdf]

Trends in prevalence and correlates of tobacco use among school-going adolescents in Bhutan; A secondary data analysis of the 2004-2019 Global Youth Tobacco Survey

Tshewang Gyeltshen, Mahbubur Rahman

2024-06-07

Contents

```
library(tidyverse)
library(ggplot2)
library(arsenal)
library(knitr)
library(gtsummary)
library(Hmisc)
library(janitor)
library(haven)
library(survey)

gyts<- haven::read_dta("gyts_managed-2.dta")
gyts1 <- haven::as_factor(gyts)


# Define the survey design using svydesign
gyts_design <- svydesign(ids = ~psu, strata = ~stratum, weights = ~finalwgt, data = gyts1, nest = T )

age <- svytable(~age + year, design = gyts_design)
age <- round(prop.table(age, margin = 2)*100, 2)

sex <- svytable(~sex + year, design = gyts_design)
sex <- round(prop.table(sex, margin = 2)*100, 2)

grad <- svytable(~grad + year, design = gyts_design)
grad <- round(prop.table(grad, margin = 2)*100, 2)


gyts_design2 <- svydesign(ids = ~psu, strata = ~stratum, weights = ~finalwgt, data = gyts2, nest = T )

everusedtob <- svytable(~everusedtob + year, design = gyts_design2)
everusedtob <- round(prop.table(everusedtob, margin = 2)*100, 2)
#write.csv(everusedtob, "everusedtob.csv")

currenttobuser <- svytable(~currenttobuser + year, design = gyts_design2)
currenttobuser <- round(prop.table(currenttobuser, margin = 2)*100, 2)
```

```r
#write.csv(currenttobuser, "currenttobuser.csv")

currentsmkuser <- svytable(~currentsmkuser + year, design = gyts_design2)
currentsmkuser <- round(prop.table(currentsmkuser, margin = 2)*100, 2)
#write.csv(currentsmkuser, "currentsmkuser.csv")

currentsmklssuser <- svytable(~currentsmklssuser + year, design = gyts_design2)
currentsmklssuser <- round(prop.table(currentsmklssuser, margin = 2)*100, 2)
#write.csv(currentsmklssuser, "currentsmklssuser.csv")

domaever <- svytable(~domaever + year, design = gyts_design2)
domaever <- round(prop.table(domaever, margin = 2)*100, 2)
#write.csv(domaever, "domaever.csv")

currentdomauser <- svytable(~currentdomauser + year, design = gyts_design2)
currentdomauser <- round(prop.table(currentdomauser, margin = 2)*100, 2)
#write.csv(currentdomauser, "currentdomauser.csv")

# Age and Sex Stratified Weighted Prevalence of Tobacco use during the survey years
stratified <- svytable(~currenttobuser + year + age, design = gyts_design2)
stratified <- round(prop.table(stratified, margin = 2)*100, 2)

# Read the data and maintain levels as factors
btn19 <- read.dta("data_management/BHUTAN_19.dta", convert.factors = TRUE)

btn2019 <- btn19 %>%
  mutate(year = 2019) %>%
  rename(Age = BTR1,
         Sex = CR2,
         Grade = BTR3,
         Student_Type = BTR4,
         Pocket_Money = BTR5,
         smke = CR5,
         Initiation_Age = CR6,
         smke30 = CR7,
         cigprday = CR8,
         Other_Smoked_Products = CR9,
         smkeprdcts30 = CR10,
         agesmkedtob = BTR12,
         othrsmkdays = BTR13,
         mornsmke = CR11,
         smkeagain = CR12,
         smkeplce = BTR16,
         smklsstob = CR13,
         smklsstob30 = CR14,
         smklssage = SLR1,
         smklssdays = SLR2,
         freqsmklssday = SLR3,
         mornsmklss = SLR4,
         smklssagain = SLR5,
         smklssquit = SLR6,
         smklssquit12 = SLR7,
         smkelssquitwill = SLR8,
```

```r
        smklsshelp = BTR27,
        smklsstobget = BTR28,
        smklssrefage = SLR11,
        offerfriend = SLR14,
        smklssquitdiff = SLR15,
        enjysmklssop = SLR17,
        domaever =  BTR33,
        arecaprdcts = BTR35,
        doma30 = BTR34,
        arecaprdcts30 = BTR36,
        tobusereason = BTR37,
        quitwilling = CR15,
        quittry12mos = CR16,
        stopsmkattde = CR17,
        stopsmktime = OR12,
        reasonstop = BTR42,
        helpquitsmke = BTR43,
        Passive_Smoking = CR19,
        shspublic_indr = CR20,
        shspublic_otdr = CR21,
        seesmke_campus = CR22,
        Opinion_on_Passive_Smoking = CR23,
        ban_publicindr = CR24,
        Public_place_ban = CR25,
        smkaccess30 = BTR51,
        cigrefuse_age = CR27,
        cigbuy_how = CR28,
        tob_buyplace = BTR54,
        Buy_Near_School = OR22,
        accesstob_buy = OR23,
        Cost_per_10_packs = BTR57,
        Media_Promotion = CR30,
        Event_Promotion = CR31,
        warn_cigpacket = CR32,
        dangertob_clss = CR33,
        On_Screen_Tobacco_Use = CR34,
        pos_advert30 = CR35,
        Tobacco_Accessories = CR36,
        Ownership_logos =  CR37,
        Promotion_company = CR38,
        Parent_Smoking_Status = OR45,
        Bestfriend_Smoking_Status = OR46,
        See_teachers_Smoke = OR60,
        Peer_Pressure = CR39,
        tobuse12mos = CR40,
        Quit_Perception = CR41,
        Social_Comfort = CR42,
        enjycigrt_op = CR43,
        question_lang = BTR75,
        stratum = Stratum,
        psu = PSU,
        finalwgt = FinalWgt) %>%
  mutate(everusedtob = case_when(
```

```
    smke == "Yes"| Other_Smoked_Products == "Yes"|smklsstob == "Yes" ~ "YES",
    smke == "No"| Other_Smoked_Products == "No"|smklsstob == "No" ~ "NO",
    TRUE ~ NA_character_
  ),
  currenttobuser = case_when(
    smke30 %in% c("1 or 2 days", "3 to 5 days", "6 to 9 days", "10 to 19 days", "20 to 29 days", "All 30
      smklsstob30 == "Yes" ~ "YES",
    smke30 == "0 days" | smklsstob30 == "No"  ~ "NO",
    TRUE ~ NA_character_
  ),
  currentsmkuser = case_when(
    smke30 %in% c("1 or 2 days", "3 to 5 days", "6 to 9 days", "10 to 19 days","20 to 29 days", "All 30
    smke30 == "0 days" ~ "NO",
    TRUE ~ NA_character_
    ),
  currentsmklssuser = if_else(smklsstob30 == "Yes", "YES", "NO"),
  Current_DOMA_user = case_when(
    doma30 %in% c("1 or 2 days", "3 to 5 days", "6 to 9 days", "10 to 19 days", "20 to 29 days", "All 30
    doma30 == "0 days" ~ "NO",
    TRUE ~ NA_character_
  ),
  currenttobuser = if_else(currenttobuser == "YES", 1, 0),
         everusedtob= if_else(everusedtob == "YES", 1, 0),
         currentsmkuser = if_else(currentsmkuser == "YES", 1, 0),
         currentsmklssuser = if_else(currentsmklssuser=="YES", 1, 0),
         Passive_Smoking = if_else(Passive_Smoking %in% c("1 to 2 days", "3 to 4 days", "5 to 6 days",
         Opinion_on_Passive_Smoking = if_else(Opinion_on_Passive_Smoking %in% c("Definitely not", "Proba
         Peer_Pressure = if_else(Peer_Pressure %in% c("Definitely not", "Probably not"), "NO", "Yes"),
         Quit_Perception = if_else(Quit_Perception %in% c("Definitely not", "Probably not"), "NO", "Yes
  Initiation_Age = case_when(Initiation_Age  %in% c("7 years old or younger", "8 or 9 years old",  "10 o
                             Initiation_Age %in% c("12 or 13 years old", "14 or 15 years old") ~ "13-15
                             Initiation_Age %in% c( "16 years old or older") ~ "16 years old or older",
                             TRUE ~ Initiation_Age),
  Cost_per_10_packs = case_when(Cost_per_10_packs %in% c("Less than Nu. 100", "Nu. 100-150", "Nu. 151-20
                                Cost_per_10_packs %in% c("Nu. 251-300", "Nu. 301-350", "Nu. 351-400") ~
                                TRUE ~ Cost_per_10_packs),
  Bestfriend_Smoking_Status = if_else(Bestfriend_Smoking_Status == "None of them", "No", "Yes"),
  Parent_Smoking_Status = case_when(Parent_Smoking_Status %in% c("Both", "Father only", "Mother only" )
                                    Parent_Smoking_Status %in% c("None") ~ "No",
                                    TRUE ~ Parent_Smoking_Status),
  Tobacco_Accessories = if_else(Tobacco_Accessories %in% c("Yes", "Maybe"), "Yes", "No"),
  Event_Promotion = if_else(Event_Promotion == "I did not go to sports events, fairs, concerts, or commu
  Initiation_Age = if_else(Initiation_Age == "I have never tried smoking a cigarette", "Never tried", In
  Age = case_when(Age %in% c("11 years old or younger", "12 years old") ~ "12 years or younger",
                  Age %in% c("13 years old", "14 years old", "15 years old") ~ "13-15 years old",
                  Age %in% c("16 years old", "17 years old", "18 years old or older") ~ ">= 16 years"),
  Pocket_Money = case_when(Pocket_Money %in% c("Less than Nu. 100", "Nu. 100-300", "Nu. 301-500") ~ "Les
                           Pocket_Money %in% c("Nu. 501-700", "Nu. 701-1000", "Nu. 1000 and above")~ "Mo
                           Pocket_Money %in% c("I usually don't have any spending money")~ "No Pocket Mo
  Grade = case_when(Grade %in% c("Class 7", "Class 8", "Class 9") ~ "Junior High School",
                    Grade %in% c("Class 10", "Class 11") ~ "High School"),
  See_teachers_Smoke = case_when(See_teachers_Smoke %in% c("About every day", "Sometimes") ~ "Yes",
                                 TRUE ~ See_teachers_Smoke))
```

```r
btn2019 <- na.omit(btn2019)

# Convert to factors and set reference levels
btn2019$Age <- relevel(factor(btn2019$Age), ref = "12 years or younger")
btn2019$Grade <- relevel(factor(btn2019$Grade), ref = "High School")
btn2019$Pocket_Money <- relevel(factor(btn2019$Pocket_Money), ref = "No Pocket Money")
btn2019$Initiation_Age <- relevel(factor(btn2019$Initiation_Age), ref = "Never tried")
btn2019$Other_Smoked_Products <- relevel(factor(btn2019$Other_Smoked_Products), ref = "No")
btn2019$Buy_Near_School <- relevel(factor(btn2019$Buy_Near_School), ref = "No")
btn2019$Parent_Smoking_Status <- relevel(factor(btn2019$Parent_Smoking_Status), ref = "No")
btn2019$Event_Promotion <- relevel(factor(btn2019$Event_Promotion), ref = "No")
btn2019$Student_Type <- relevel(btn2019$Student_Type, ref = "Boarding student")
btn2019$Media_Promotion <- relevel(factor(btn2019$Media_Promotion), ref = "No")
btn2019$Passive_Smoking <- relevel(factor(btn2019$Passive_Smoking), ref = "Not Exposed")
btn2019$Social_Comfort <- relevel(factor(btn2019$Social_Comfort), ref = "No difference whether smoking o
btn2019$See_teachers_Smoke <- relevel(factor(btn2019$See_teachers_Smoke), ref = "Never")

#Fitting Uni-variate Logistic regression
gyts_design <- svydesign(ids = ~psu, strata = ~stratum, weights = ~finalwgt, data = btn2019, nest = T )

Age <- svyglm(currenttobuser ~  Age,  gyts_design, family = quasibinomial())
Sex <- svyglm(currenttobuser ~  Sex,  gyts_design, family = quasibinomial())
Pocket_Money <- svyglm(currenttobuser ~  Pocket_Money,  gyts_design, family = quasibinomial())
Student_Type<- svyglm(currenttobuser ~  Student_Type,  gyts_design, family = quasibinomial())
Initiation_Age<- svyglm(currenttobuser ~  Initiation_Age,  gyts_design, family = quasibinomial())
Other_Smoked_Products <- svyglm(currenttobuser ~  Other_Smoked_Products,  gyts_design, family = quasibin
Passive_Smoking <- svyglm(currenttobuser ~  Passive_Smoking,  gyts_design, family = quasibinomial())
On_Screen_Tobacco_Use <- svyglm(currenttobuser ~  On_Screen_Tobacco_Use,  gyts_design, family = quasibin
Tobacco_Accessories<- svyglm(currenttobuser ~  Tobacco_Accessories,  gyts_design, family = quasibinomial
Cost_per_10_packs<- svyglm(currenttobuser ~  Cost_per_10_packs,  gyts_design, family = quasibinomial())
Media_Promotion<- svyglm(currenttobuser ~  Media_Promotion,  gyts_design, family = quasibinomial())
Event_Promotion<- svyglm(currenttobuser ~  Event_Promotion,  gyts_design, family = quasibinomial())
dangertob_clss<- svyglm(currenttobuser ~  dangertob_clss,  gyts_design, family = quasibinomial())
Ownership_logos<- svyglm(currenttobuser ~  Ownership_logos,  gyts_design, family = quasibinomial())
Peer_Pressure<- svyglm(currenttobuser ~  Peer_Pressure,  gyts_design, family = quasibinomial())
Quit_Perception<- svyglm(currenttobuser ~  Quit_Perception,  gyts_design, family = quasibinomial())
Social_Comfort<- svyglm(currenttobuser ~  Social_Comfort,  gyts_design, family = quasibinomial())
Buy_Near_School<- svyglm(currenttobuser ~  Buy_Near_School,  gyts_design, family = quasibinomial())
Parent_Smoking_Status<- svyglm(currenttobuser ~  Parent_Smoking_Status,  gyts_design, family = quasibino
Bestfriend_Smoking_Status<- svyglm(currenttobuser ~  Bestfriend_Smoking_Status,  gyts_design, family = q
See_teachers_Smoke<- svyglm(currenttobuser ~  See_teachers_Smoke,  gyts_design, family = quasibinomial()
Current_DOMA_user<- svyglm(currenttobuser ~  Current_DOMA_user,  gyts_design, family = quasibinomial())
Grade<- svyglm(currenttobuser ~  Grade,  gyts_design, family = quasibinomial())

# Step-wise-Multivariate Analysis - Forward
null2019 <- glm(currenttobuser ~ 1, data = btn2019, family = binomial(link = "logit"))
full2019 <- glm(currenttobuser ~
                  Age +
                  Sex +
                  Pocket_Money + #pocket money
                  Initiation_Age + # Age when first cigarette was taken
                  Other_Smoked_Products + #tried other smoked products other than cigarretes
                  Passive_Smoking + #Exposure to secondhand smoke at home
```

```
                  #Opinion_on_passive_smoking #Opinion on secondhand Smoking
                  Cost_per_10_packs + #Payment for 10 pack of cigarettes
                  Media_Promotion + #Anti-tobacco media messages in past 30 days
                  Event_Promotion + #Anti-tobacco messages in events
                  dangertob_clss + #Danger of tobacco use taught in class in last 1 year
                  On_Screen_Tobacco_Use + #See  people using tobacco on TV or movies in last 30 days
                  Tobacco_Accessories + #Wear accessories with tobacco product name or picture
                  Ownership_logos + #Tobacco product logo
                  Peer_Pressure +
                  Quit_Perception + #Think it is difficult to quit after smoking
                  Social_Comfort + #Social gathering comfortability with smoking
                  Student_Type + #Student type, boarders or day scholar
                  Buy_Near_School + #Available to buy the tobacco products near the school
                  Parent_Smoking_Status + #Parents Smoking Status
                  Bestfriend_Smoking_Status + #Best friend's smoking status
                  See_teachers_Smoke +
                  Current_DOMA_user+ #See teachers smoking outdoor or in school
                Grade,
                data = btn2019, family = binomial(link = "logit"))

forward2019<- step(null2019, direction = "forward", scope = formula(full2019), trace = 0)

car::vif(full2019)

forward2019$aic

tbl_regression(forward2019, exponentiate = T)


library(sjPlot)
library(ggplot2)

p <- plot_model(
  forward2019,
  title = "Effect Estimates of each Correlates, Global Youth Tobacco Survey, 2004 - 2019, Bhutan",
  wrap.title = 100,
  axis.title = "Risk Ratios",
  type = "std",
  sort.est = TRUE,
  vline.color = "purple"
)

# Customize the plot with a theme
p + theme_bw() +
  theme(
    plot.title = element_text(hjust = 0.5),  # Center-align the title
    axis.text = element_text(size = 12),     # Adjust text size for readability
    axis.title = element_text(size = 14)     # Adjust axis title size
  )

#Visualization
# Filter out rows with NAs in the "Sex" variable
gyts2_filtered <- gyts2 %>%
```

```
  filter(!is.na(sex))

# Collapsing data by year for summary and graphs
prevalencetab <- gyts2_filtered %>%
  group_by(year, sex) %>%
  summarise(
    count_everusedtob = sum(everusedtob == "YES", na.rm = TRUE),
    total_everusedtob = sum(!is.na(everusedtob)),
    count_currenttobuser = sum(currenttobuser == "YES", na.rm = TRUE),
    total_currenttobuser = sum(!is.na(currenttobuser)),
    count_currentsmkuser = sum(currentsmkuser == "YES", na.rm = TRUE),
    total_currentsmkuser = sum(!is.na(currentsmkuser)),
    count_currentsmklssuser = sum(currentsmklssuser == "YES", na.rm = TRUE),
    total_currentsmklssuser = sum(!is.na(currentsmklssuser)),
    count_currentdomauser = sum(currentdomauser == "YES", na.rm = TRUE),
    total_currentdomauser = sum(!is.na(currentdomauser)),
    count_domaever = sum(domaever == "YES", na.rm = TRUE),
    total_domaever = sum(!is.na(domaever)),
    count_susceptible = sum(susceptiblepop == "YES", na.rm = TRUE),
    total_susceptible = sum(!is.na(susceptiblepop))
  ) %>%
  mutate(
    everusedtob_prev = count_everusedtob / total_everusedtob * 100,
    currentuse_prev = count_currenttobuser / total_currenttobuser * 100,
    currntsmke_prev = count_currentsmkuser / total_currentsmkuser * 100,
    currntsmklss_prev = count_currentsmklssuser / total_currentsmklssuser * 100,
    currentdoma_prev = count_currentdomauser / total_currentdomauser * 100,
    everuseddoma_prev = count_domaever / total_domaever * 100,
    susceptible = count_susceptible / total_susceptible * 100
  )

# Tobacco use plot faceted by sex with data labels, loess smooth, point shape 15, and nudged 2019 value
prevalencetab %>%
  ggplot(., aes(x = year)) +
  geom_point(aes(y = everusedtob_prev, color = "Ever Used"), shape = 15, size = 3) +
  geom_point(aes(y = currentuse_prev, color = "Current Users"), shape = 15, size = 3) +
  geom_point(aes(y = currntsmke_prev, color = "Current Smokers"), shape = 15, size = 3) +
  geom_point(aes(y = currntsmklss_prev, color = "Current Smokeless Users"), shape = 15, size = 3) +
  geom_smooth(aes(y = everusedtob_prev, color = "Ever Used"), method = "loess", se = FALSE, size = 1) +
  geom_smooth(aes(y = currentuse_prev, color = "Current Users"), method = "loess", se = FALSE, size = 1
  geom_smooth(aes(y = currntsmke_prev, color = "Current Smokers"), method = "loess", se = FALSE, size =
  geom_smooth(aes(y = currntsmklss_prev, color = "Current Smokeless Users"), method = "loess", se = FAL
  geom_text(aes(y = everusedtob_prev, label = round(everusedtob_prev, 1)), vjust = -0.5, size = 3) +
  geom_text(aes(y = currentuse_prev, label = round(currentuse_prev, 1)), vjust = -0.5, size = 3) +
  geom_text(aes(y = currntsmke_prev, label = round(currntsmke_prev, 1)), vjust = -0.5, size = 3,
            data = subset(prevalencetab, !(sex == "Female" & year == 2019))) +
  geom_text(aes(y = currntsmke_prev, label = round(currntsmke_prev, 1)), vjust = -0.5, size = 3,
            nudge_x = 0.00, nudge_y = 0.01, data = subset(prevalencetab, sex == "Female" & year == 2019
  geom_text(aes(y = currntsmklss_prev, label = round(currntsmklss_prev, 1)), vjust = -0.5, size = 3,
            data = subset(prevalencetab, !(sex == "Female" & year == 2019))) +
  geom_text(aes(y = currntsmklss_prev, label = round(currntsmklss_prev, 1)), vjust = -0.5, size = 3,
            nudge_x = -0.00, nudge_y = -0.07, data = subset(prevalencetab, sex == "Female" & year == 20
  labs(
```

```r
    x = "GYTS Survey Years",
    y = "Weighted Prevalence in Percentage",
    title = "Tobacco Use Trends among students by Sex in Bhutan (2004-2019)",
    caption = "Data Source: Global Youth Tobacco Survey, Bhutan"
  ) +
  scale_x_continuous(breaks = unique(prevalencetab$year)) +
  theme_minimal() +
  theme(
    plot.title = element_text(size = 16, face = "bold", hjust = 0.5),
    axis.text.x = element_text(angle = 45, hjust = 1, size = 12),
    axis.text.y = element_text(size = 12),
    legend.title = element_blank(),
    legend.text = element_text(size = 12),
    legend.position = "top",
    plot.caption = element_text(hjust = 0.99, size = 10, face = "italic"),
    strip.text = element_text(size = 12, face = "bold")
  ) +
  facet_wrap(~ sex, ncol = 2, scales = "free_x")

# Tobacco use plot faceted by sex with data labels, point shape 15, and nudged 2019 values for females
prevalencetab %>%
  ggplot(., aes(x = year)) +
  geom_point(aes(y = everusedtob_prev, color = "Ever Used"), shape = 15, size = 2) +
  geom_point(aes(y = currentuse_prev, color = "Current Users"), shape = 15, size = 2) +
  geom_point(aes(y = currntsmke_prev, color = "Current Smokers"), shape = 15, size = 2) +
  geom_point(aes(y = currntsmklss_prev, color = "Current Smokeless Users"), shape = 15, size = 2) +
  geom_line(aes(y = everusedtob_prev, color = "Ever Used"), size = 1) +
  geom_line(aes(y = currentuse_prev, color = "Current Users"), size = 1) +
  geom_line(aes(y = currntsmke_prev, color = "Current Smokers"), size = 1) +
  geom_line(aes(y = currntsmklss_prev, color = "Current Smokeless Users"), size = 1) +
  geom_text(aes(y = everusedtob_prev, label = round(everusedtob_prev, 1)), vjust = -0.5, size = 3) +
  geom_text(aes(y = currentuse_prev, label = round(currentuse_prev, 1)), vjust = -0.5, size = 3) +
  geom_text(aes(y = currntsmke_prev, label = round(currntsmke_prev, 1)), vjust = -0.5, size = 3,
            data = subset(prevalencetab, !(sex == "Female" & year == 2019))) +
  geom_text(aes(y = currntsmke_prev, label = round(currntsmke_prev, 1)), vjust = -0.5, size = 3,
            nudge_x = 0.00, nudge_y = 0.01, data = subset(prevalencetab, sex == "Female" & year == 2019
  geom_text(aes(y = currntsmklss_prev, label = round(currntsmklss_prev, 1)), vjust = -0.5, size = 3,
            data = subset(prevalencetab, !(sex == "Female" & year == 2019))) +
  geom_text(aes(y = currntsmklss_prev, label = round(currntsmklss_prev, 1)), vjust = -0.5, size = 3,
            nudge_x = -0.00, nudge_y = -0.07, data = subset(prevalencetab, sex == "Female" & year == 20
  labs(
    x = "GYTS Survey Years",
    y = "Weighted Prevalence in Percentage",
    title = "Tobacco Use Trends among students by Sex in Bhutan (2004-2019)",
    caption = "Data Source: Global Youth Tobacco Survey, Bhutan"
  ) +
  scale_x_continuous(breaks = unique(prevalencetab$year)) +
  theme_minimal() +
  theme(
    plot.title = element_text(size = 16, face = "bold", hjust = 0.5),
    axis.text.x = element_text(angle = 45, hjust = 1, size = 12),
    axis.text.y = element_text(size = 12),
    legend.title = element_blank(),
```

```
    legend.text = element_text(size = 12),
    legend.position = "top",
    plot.caption = element_text(hjust = 0.99, size = 10, face = "italic"),
    strip.text = element_text(size = 12, face = "bold")
  ) +
  facet_wrap(~ sex, ncol = 2, scales = "free_x")


for (i in 1:nrow(prevalencetab)){
  for (j in 17:20){
      prevalencetab[i,j] <- log(prevalencetab[i,j])
      }
  }


# Calculate Average Annual Change percentage
library(jtools)
prevalencetab2 <- gyts2_filtered %>%
  group_by(year) %>%
  summarise(
    count_everusedtob = sum(everusedtob == "YES", na.rm = TRUE),
    total_everusedtob = sum(!is.na(everusedtob)),
    count_currenttobuser = sum(currenttobuser == "YES", na.rm = TRUE),
    total_currenttobuser = sum(!is.na(currenttobuser)),
    count_currentsmkuser = sum(currentsmkuser == "YES", na.rm = TRUE),
    total_currentsmkuser = sum(!is.na(currentsmkuser)),
    count_currentsmklssuser = sum(currentsmklssuser == "YES", na.rm = TRUE),
    total_currentsmklssuser = sum(!is.na(currentsmklssuser))
  ) %>%
  mutate(
    everused_prev = count_everusedtob/total_everusedtob * 100,
    currentuse_prev = count_currenttobuser / total_currenttobuser * 100,
    currntsmke_prev = count_currentsmkuser / total_currentsmkuser * 100,
    currntsmklss_prev = count_currentsmklssuser / total_currentsmklssuser * 100
  )

aapcdata %>%
  View()
# Create a frame with year and prevalence data
aapcdata <- data.frame(
  year = prevalencetab2$year,
  everused = prevalencetab2$everused_prev,
  currentuse = prevalencetab2$currentuse_prev,
  smokers = prevalencetab2$currntsmke_prev,
  smkeless = prevalencetab2$currntsmklss_prev
)

colnames(aapcdata) <- c("years", "ever_used", "current_tob_users", "current_smokers", "current_smokeles

# Calculate the Average Annual Prevalence Change (AAPC) for everused
fit <- lm(log(current_tob_users) ~ years + I(years^2), data = aapcdata)

# Take log of prevalence
```

```
aapcdata$log_everused <- log(aapcdata$ever_used)
aapcdata$log_tob <- log(aapcdata$current_tob_users)
aapcdata$log_smoke <- log(aapcdata$current_smokers)
aapcdata$log_smokeless <- log(aapcdata$current_smokeless)

# Linear regression
fit_everused <- lm(log_everused ~ years + years^2, data=aapcdata)
fit_tob <- lm(log_tob ~ years + years^2, data=aapcdata)
fit_smoke <- lm(log_smoke ~ years + years^2, data=aapcdata)
fit_smokeless <- lm(log_smokeless ~ years + years^2, data=aapcdata)

summary(fit_everused)
# Get coefficients
beta_everused <- coef(fit_everused)[2]
beta_tob <- coef(fit_tob)[2]
beta_smoke <- coef(fit_smoke)[2]
beta_smokeless <- coef(fit_smokeless)[2]

# Calculate AAPC
AAPC_everused <- (exp(beta_everused) - 1) * 100
AAPC_tob <- (exp(beta_tob) - 1) * 100
AAPC_smoke <- (exp(beta_smoke) - 1) * 100
AAPC_smokeless <- (exp(beta_smokeless) - 1) * 100

# Print results
print(AAPC_everused)
print(AAPC_tob)
print(AAPC_smoke)
print(AAPC_smokeless)

prevfinaltab <- data.frame(aapcdata$years, aapcdata$ever_used, aapcdata$current_tob_users, aapcdata$cur[illegible]
```
