# Supplementary material for: Trends in prevalence and correlates of tobacco use among school-going adolescents in Bhutan: A secondary data analysis of the 2004–2019 Global Youth Tobacco Survey
Source: PLOS Glob Public Health. 2024 Jul 26;4(7):e0003544. doi: 10.1371/journal.pgph.0003544 (PMC11280154; doi:10.1371/journal.pgph.0003544)
Supplement: Attachment — Submitted filename: Response to Reviewers.docx [file pgph.0003544.s002.docx]

Dear Reviewers,

Thank you very much for your careful review and insightful comments on this manuscript. Your feedback has been invaluable, and I believe the revised manuscript is significantly improved because of your suggestions.

Your comments and suggestions have been incorporated where appropriate.

We remain thankful for your time.

Authors

Journal Requirements:

1. Please amend your online Financial Disclosure statement. If you did not receive any funding for this study, please simply state: “The authors received no specific funding for this work.”

2. Please update your online Competing Interests statement. If you have no competing interests to declare, please state: “The authors have declared that no competing interests exist.”

3. Please provide separate figure files in .tif or .eps format only and remove any figures embedded in your manuscript file. Please also ensure that all files are under our size limit of 10MB. You may leave the figure captions or legends in the manuscript.

Reviewers' comments:

Reviewer's Responses to Questions

Comments to the Author

1. Does this manuscript meet PLOS Global Public Health’s publication criteria? Is the manuscript technically sound, and do the data support the conclusions? The manuscript must describe methodologically and ethically rigorous research with conclusions that are appropriately drawn based on the data presented.

Reviewer #1: Yes

Reviewer #2: Partly

2. Has the statistical analysis been performed appropriately and rigorously?

Reviewer #1: Yes

Reviewer #2: Yes

3. Have the authors made all data underlying the findings in their manuscript fully available (please refer to the Data Availability Statement at the start of the manuscript PDF file)?

The PLOS Data policy requires authors to make all data underlying the findings described in their manuscript fully available without restriction, with rare exception. The data should be provided as part of the manuscript or its supporting information, or deposited to a public repository. For example, in addition to summary statistics, the data points behind means, medians and variance measures should be available. If there are restrictions on publicly sharing data—e.g. participant privacy or use of data from a third party—those must be specified.

Reviewer #1: Yes

Reviewer #2: Yes

4. Is the manuscript presented in an intelligible fashion and written in standard English?

PLOS Global Public Health does not copyedit accepted manuscripts, so the language in submitted articles must be clear, correct, and unambiguous. Any typographical or grammatical errors should be corrected at revision, so please note any specific errors here.

Reviewer #1: Yes

Reviewer #2: Yes

5. Review Comments to the Author

Please use the space provided to explain your answers to the questions above. You may also include additional comments for the author, including concerns about dual publication, research ethics, or publication ethics. (Please upload your review as an attachment if it exceeds 20,000 characters)

Reviewer #1: This is a very important research work. However, the authors should take note of the following.

**General comments**

GYTS is designed to be representative of youths 13-15 years, but the authors did not specify why the decided to focus on a different age cohort. Moreover, it is difficult for readers to know which age cohort was used as there is no consistency in the use of the age cohort included in the analysis. Sometimes you use 11-17, 11-18 and 11-19 years and line 22 indicates under 11 years, what was the minimum and maximum age?

Thank you for your comments.

We apologize for any confusion caused by the inconsistent use of age cohorts in our study. The GYTS is designed to be representative of youths aged 13-15 years. However, due to the school-based sampling frame, the survey has included students who are outside of this representative age range. The questionnaire asks respondents to choose from the following age categories:

a) 11 years old or younger,

b) 12 years old,

c) 13 years old,

d) 14 years old,

e) 15 years old,

f) 16 years old, and

g) 17 years old or older/18 years old or older.

While the minimum age is consistently set to “11 years or younger” across survey years, the maximum age varies, with some surveys using “17 years or older,” “18 years or older,” or “19 years or older.” As age data was collected as a categorical variable, we cannot ascertain the exact minimum and maximum ages of respondents beyond the lower and upper limits set in the survey questionnaire.

To accommodate these inconsistencies and to include all surveyed individuals, including those beyond the representative age of 13-15 years, we have used varying upper age limits in our study.

Why was the term adolescent used instead of youth since you are using GYTS data? I understand adolescent covers the ages included in your study, but it is better to be consistent with the GYTS study protocol.

We chose to use the term “adolescent” instead of “youth” because, according to the World Health Organization's definition, adolescents are individuals aged 10-19 years. Our study fully covers this age group, whereas the term “youth” may include individuals older than 19 years. Furthermore, as our study includes age groups outside of the GYTS representative age range of 13-15 years old, we believe that the term “adolescent” is more appropriate than “youth.”

Similar comments have been made by the second reviewer suggesting the use of term students instead. We submit the revised manuscript incorporating the suggestion to use “students”.

Please take note of the spelling and grammatical errors. Consider getting the services of a native English speaker to proofread the document.

Thank you for your attention. In the revised version, we have made efforts to minimize errors as much as possible.

Specific comments

**Introduction**

Line 45- 49 -Do you mean tobacco use was banned? Did enactment of the Tobacco Control Act and enforcement of the ban occur at the same time? The sequence is not clear and might be confusing to readers.

Line 53-55, report the actual prevalence and mention the year.

Line 45-49: Revised the sentence to have more clarity flow. “. In June 2010 the first democratically elected government of Bhutan enacted the Tobacco Control Act, which was followed by the enforcement of a comprehensive nationwide ban on tobacco use in the same year”.

Line 53-55

Revised as suggested to mention the year and prevalence rate.

**Methods**

Line 62 – Mention the total number of time points and survey years.

Revised as suggested.

Line 77-78- Revise the sentences.

The sentence has been revised to incorporate survey years and time and syntax errors.

Line 83-85- Reframe this sentence “The outcome variable for the study was tobacco use- defined as use of any form of tobacco (smoked/smokeless) currently using at the time of the survey.”

Revised to define the outcome variables as suggested. [Line:84-94]

Can you please define the key variables used in your analysis?

Key variables of interest have been described as suggested. [121-127]

Specify if all the surveys included in your analysis were national or subnational surveys.

Revision made: Added the information in line 120-121.

GYTS uses a complex survey design approach, was this controlled for in the analysis? I understand you mentioned in lines 109-110 that a weighted analysis was conducted but you did not specify what the weighting controlled for. Why did you not report standard errors and/or confidence intervals to enable readers assess the precision of your point estimates?

Thank you for your attention to this important point. It is true that GYTS uses complex survey design approaches. To account for the complex survey design of the GYTS, we used the survey package in R. Using this package, we created a survey design object with the following specifications: primary sampling units (PSUs) were specified using the ids argument, stratification was incorporated using the strata argument, and the final survey weights were applied using the weights argument. This approach allowed us to properly account for the nested structure of the data and ensure accurate variance estimation. This has been clearly explained in revised manuscript.

Supplemental Files with reproducible R codes will be provided in the revised submission for interested readers who may be interested in the precision of estimates.

**Results**

Lines 143 to 144 Revise this sentence: “The prevalence of tobacco uses “ever used".

tobacco”, “current use”, “smoked”, and “smokeless” are presented separately.”

Sentence revised:
The prevalence *of* “ever used tobacco” *and* “current use” *(both smoked and smokeless)*, “smoked”, and “smokeless” *tobacco use* are presented.

Lines 158-159: The sentence is difficult to understand, consider revising it.

Revised the sentence as suggested [Line169-172].

Lines 177 to 179 -How was first tobacco exposure before 11 years defined.

The “age of Exposure” was a derived composite variable from questions; *“How old were you when you first tried a cigarette?”*, *“How old were you when you first tried using smokeless tobacco?”*, and *“How old were you when you first used any form of smoked tobacco products other than cigarettes”. All these questions had response labels as follow:*

1 I have never smoked cigarettes

2 7 years old or younger

3 8 or 9 years old

4 10 or 11 years old

5 12 or 13 years old

6 14 or 15 years old

7 16 years old or older

The levels have been regrouped into three levels consisting of “never tried”, **“*11 years old or younger”,*** and “13-16 years old or older”.

Line 190 the GYTS suggestion to use the term “exposure to second-hand smoke” instead of “passive smoking” as this is the term used in the GYTS tool.

Revision made as suggested.

**Discussion**

Lines 201-203 (first and second sentences) do not fit here and should be deleted.

Revision made as suggested.

**Reviewer #2: General comments:**

For the sake of clarity and precision, I strongly recommend using the term 'students' instead of 'youth' in your manuscript. This is particularly important as the GYTS is a nationally representative survey of students in public and private schools, and it does not encompass all youth, especially those not educated in formal settings.

Thank you for your comments and suggestions. We understand the need for clarity in use of terms to either youth/adolescent or students. Similar comments were made by the first reviewer. Upon careful consideration, we have revised the manuscript using the term “students” where necessary in the revised manuscript. We thank you for this suggestion.

The methods section did not include the measures /definition used for smoked and smokeless tobacco. At a minimum, definitions for current cigarette smoking, current other smoked tobacco products, and current smokeless tobacco use should be provided. Current and ever use are not defined in the manuscript but are used throughout.

Thank you for your comments and suggestions. In the revision, the outcome variable has been defined and how they have been derived has been mentioned. The definition and constituent questions used to derive the composite variable have been mentioned. [Line 84-94]

Given that GYTS targets students aged 13-15 and the chosen analytic sample is heavily weighted toward this population group, I suggest focusing your analysis on this age range. Not doing so may bias your findings toward this age group.

Thank you for your insightful comments and suggestions. We appreciate the importance of focusing on the 13-15 age range, as targeted by the GYTS. However, our objective is to provide a broader overview of adolescent tobacco use prevalence trends, not limited to just the 13-15 years age group. We believe that analysing the general adolescent population will offer a more comprehensive understanding of tobacco use patterns and trends across different age groups within the adolescent category.

To address potential bias, we have taken measures to ensure that our analysis accurately reflects the distribution of the sample and appropriately weights the data. We believe that this approach will provide valuable insights and contribute to a more holistic view of adolescent tobacco use.

The manuscript needs editorial review to address minor grammatical issues throughout the paper.

Thank you for your attention. In the revised version, we have made efforts to minimize errors as much as possible.

Introduction

Line 32: Suggest using tobacco use instead of “uses.”

Amendments made as suggested.

Line 33: Suggest saying “nicotine addiction” or “nicotine dependence” instead of addiction.

Amendments made as suggested.

Lines 33-35: Suggest using the latest estimate included in the most recent WHO global trends report (WHO global report on trends in prevalence of tobacco use 2000–2030. Geneva: World Health Organization; 2024.). For line 35, I recommend using the estimate for tobacco use, not smoking, given that your manuscript is focused on tobacco use.

Thank you for very insightful comment. The manuscript has been revised as per the suggestions and necessary citation has been cited. [Line 35, ref 2]

Line 36: Please add a comma after “world.”
Amendments made as suggested.

Line 37: Please capitalize the first letters in the global youth tobacco survey.

Amendments made as suggested.

Line 44: Spell out WHO since it's your first time using the initials.

Amendments made as suggested.

Line 51: The GYTS is part of the Global Tobacco Surveillance System, which includes multiple partners, such as the US Centers for Disease Control, which provides technical assistance. Given the partnership, I suggest not singling out WHO here.

Thank you for your suggestion. I believe you meant Line 61. In the revision, the word WHO has been omitted and only Global Youth Tobacco Survey has been retained. Thank you.

Methods

Line 67-68: Please add a citation for the following statement: “Bhutan is also known internationally for its measure of 68 Gross National Happiness as a development Index.”

Thank you for the comment. This sentence and the subsequent sentence have been merged and citation provided in the revised manuscript.

Line 72-75: The authors should include a justification for why their analytic sample included students aged 11-18 when GYTS is designed to target students aged 13-15.

Thank you for the comment. Similar comments were also made by the first reviewer. The GYTS is designed to be representative of youths aged 13-15 years. However, due to the school-based sampling frame, the survey has included students who are outside of this representative age range.

While the minimum age is consistently set to “11 years or younger” across survey years, the maximum age varies, with some surveys using “17 years or older,” “18 years or older,” or “19 years or older.” Out study objective, while using the data from GYTS is to show the prevalence on whole adolescent age group and hence included all the available age groups where the dataset allowed. In the revision, this has been justified. [Line: 73-75]

Line 76-79: This paragraph can be deleted as it does not add much to the manuscript given that the public was not involved.

Thank you for the suggestion. This section was added to fulfil the journal requirement needing to declare the involvement of patient and public in the study.

Line 81: Suggest removing “WHO” per my comment regarding line 51.

Amendments made as suggested.

Line 83-85: “Current use” in GYTS is defined as use within the past 30 days, not necessarily use at the time of the survey. Please revise this statement.

Thank you very much for the suggestion. The variables have been defined clearly in the revised manuscript as per the suggestion given by the first reviewer as well.

Line 100-101: Please provide the citation for this website.

Thank you very much for the suggestion. The website for CDC has been cited in the revised manuscript.

Line 106-108: Please indicate which R package was used to conduct the analysis, if any.

Thank you very much for the suggestion. Multiple R packages were used for data management and subsequent analysis. This has been provided as a supplemental file.

Line 199: Please indicate what you will be adjusting/controlling for in your model.

Thank you for your comments, as we can see from the regression output provided in table3, the outcome variable (use of tobacco) has been adjusted for several key variables to ensure the robustness of our findings. The adjusted best-fit model includes the following covariates:

- Age of exposure
- Current use of DOMA
- Peer pressure
- Trying other smoking products
- Cost per 10 packets of cigarettes
- Best friend’s smoking status
- Ability to buy tobacco near school
- Secondhand smoking at home
- Parents' smoking status
- Exposure to tobacco logos/print
- Event promotion
- Type of student (boarding vs. day scholar)
- Age of the survey respondents
- Grade of the student
- Observing teachers smoking
- Media promotion

These adjustments were made to control for the influence of these factors on the outcomes. We hope this clarifies our approach and the considerations made in our analysis. We thank you for your attention.

Results

Line 130-131: The methods section did not describe exposure to secondhand smoke as an outcome. Why is it being reported in the results? Clarifying all measures and their definition in the methods section would be good.

Thank you for pointing this out. We would like to clarify that the mention of second-hand smoke exposure in the results section refers to the proportions of students reported to have been exposed, as described in Table 1. However, we understand that this may cause confusion. To improve clarity and flow, we revised the methods section to explicitly define all outcome variables. We also revised the flow of sentence in the results regarding second-hand smoke for better readability and coherence.

Line 140: given the target age from GYTS is 13-15, I suggest recognizing the age groups as ≤12 years; 13-15; ≥16 years.

Thank you very much for the suggestion. We agree with your suggestion that splitting age into ≤12 years; 13-15; ≥16 years makes more sense especially in this study context. We have made amendments as per your suggestion incorporating changes in both table1 and table3. Thank you very much for this insightful comment.

Line 156: The authors used sex throughout the paper but proceeded to use gender here. Please be consistent with sex and gender, as they are not interchangeable.

Thank you very much for the suggestion. We made the revision to sex in the revised manuscript.

Line 159-160: The authors mentioned that smokeless tobacco use increased two-fold (AAPC) compared with smokers throughout the survey years. This statement is misleading without knowing the percentage for the latter. Please include the percentage in parentheses.

Thank you very much for the suggestion. This has been revised in the new manuscript reflecting change percentage of the current smokers as well.

Line 161: Figure 1 is not easy to understand as it is difficult to understand the provided. Are the solid lines males and dotted line females? I suggest recreating the graphic to make it easier for readers to understand.

Thank you very much for the suggestion. The Figure one has been recreated to reflect separate graphs for male and female faced in one plot. Thank you for pointing this out.

Discussion

Lines 262-267: A significant study limitation is that the initial GYTS core questionnaire from 1999 was updated in 2012. The wording for some questions may have changed, likely affecting how students responded in subsequent surveys. Also, the findings for prevalence change might be influenced by the periodicity in which Bhutan conducted the GYTS survey, as the interval between rounds was two years, three years, four years, and six years, respectively.

Thank you very much for the suggestion. In the revision, we made sure to point this limitation out. Thank you for pointing this out in detail. [Line: 289-293]
